# Anammox Bacterial *S*-Adenosyl-l-Methionine Dependent Methyltransferase Crystal Structure and Its Interaction with Acyl Carrier Proteins

**DOI:** 10.3390/ijms24010744

**Published:** 2023-01-01

**Authors:** Tesshin Uegaki, Taisei Takei, Shuhei Yamaguchi, Keisuke Fujiyama, Yusuke Sato, Tomoya Hino, Shingo Nagano

**Affiliations:** 1 Department of Chemistry, Graduate School of Engineering, Tottori University, 4-101 Koyama-cho Minami, Tottori 680-8552, Japan; 2 Department of Chemistry and Biotechnology, Faculty of Engineering, Tottori University, 4-101 Koyama-cho Minami, Tottori 680-8552, Japan; 3 Department of Engineering, Graduate School of Sustainability Science, Tottori University, 4-101 Koyama-cho Minami, Tottori 680-8552, Japan; 4 Center for Research on Green Sustainable Chemistry, Tottori University, 4-101 Koyama-cho Minami, Tottori 680-8552, Japan

**Keywords:** anammox, ladderane lipid, fatty acid biosynthesis, methyltransferase, AlphaFold

## Abstract

Ladderane lipids (found in the membranes of anaerobic ammonium-oxidizing [anammox] bacteria) have unique ladder-like hydrophobic groups, and their highly strained exotic structure has attracted the attention of scientists. Although enzymes encoded in type II fatty acid biosynthesis (FASII) gene clusters in anammox bacteria, such as *S*-adenosyl-l-methionine (SAM)-dependent enzymes, have been proposed to construct a ladder-like structure using a substrate connected to acyl carrier protein from anammox bacteria (AmxACP), no experimental evidence to support this hypothesis was reported to date. Here, we report the crystal structure of a SAM-dependent methyltransferase from anammox bacteria (AmxMT1) that has a substrate and active site pocket between a class I SAM methyltransferase-like core domain and an additional α-helix inserted into the core domain. Structural comparisons with homologous SAM-dependent *C*-methyltransferases in polyketide synthase, AmxACP pull-down assays, AmxACP/AmxMT1 complex structure predictions by AlphaFold, and a substrate docking simulation suggested that a small compound connected to AmxACP could be inserted into the pocket of AmxMT1, and then the enzyme transfers a methyl group from SAM to the substrate to produce branched lipids. Although the enzymes responsible for constructing the ladder-like structure remain unknown, our study, for the first time, supports the hypothesis that biosynthetic intermediates connected to AmxACP are processed by SAM-dependent enzymes, which are not typically involved in the FASII system, to produce the ladder-like structure of ladderane lipids in anammox bacteria.

## 1. Introduction

Biological membranes constitute the boundaries of cells and separate cellular compartments [1]. The main components of biological membranes are various lipids, which form a bilayer structure. The most important functional feature of membranes is their low permeability, which prevents substances produced inside the membrane compartment from leaking out and prevents unwanted external molecules from diffusing in. Owing to this low permeability, the ion concentration gradient between the external and internal regions of the membrane is maintained, and for example, the proton gradient is able to then drive ATP synthesis in most organisms [2].

**Figure 1:**
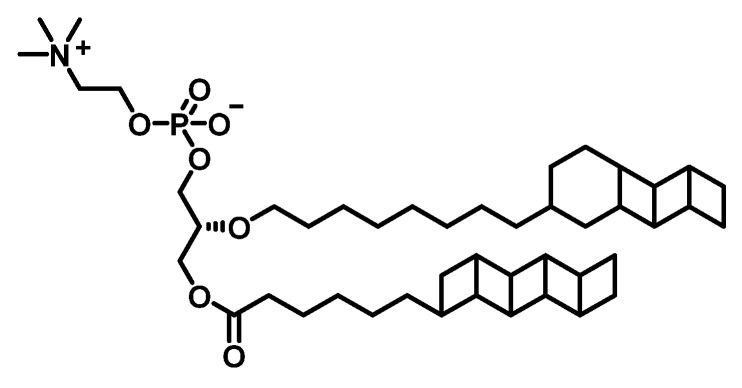
Structure of one of the most common ladderane lipids [5]-[3]-ladderane phosphatidylcholine, which is an abundantly and commonly found ladderane lipid in anammox bacteria.

Anammox bacteria oxidize relatively inert ammonia by using nitrate under anaerobic conditions (anammox reaction) to produce molecular nitrogen [3,4,5,6]. The anammox reaction, which occurs in the major cell compartment “anammoxosome” is thought to be coupled with the formation of a proton concentration gradient across the anammoxosome membrane that drives ATP synthesis [4,5,6,7,8]. Anammox bacteria have unique ladderane lipids with highly distorted hydrophobic groups consisting of three or five fused cyclobutene rings (Figure 1) [4,6,9,10]. Anammoxosome membranes consist mainly of ladderane lipids, although the distribution of ladderane lipids in anammox bacteria is not confined to anammoxosome membranes [9]. Anammox bacterial growth is slow (doubling time: 2–14 days) and, concomitantly, the proton gradient formation rate is also low [4,6,11]. Thus, low proton permeability in a regular membrane can disrupt the proton gradient. The dominant presence of ladderane lipids in the anammoxosome membranes creates considerably low proton permeability and maintains the proton gradient that drives ATP synthesis in the anammoxosome membrane [10].

Three or five linearly concatenated cyclobutene rings that are found in ladderane lipids are not found in other natural products. Although several chemical synthetic routes for producing ladder-like skeletons have been reported, the chemical creation of a highly distorted ladder-like moiety remains difficult and challenging [12,13]. Therefore, the biosynthetic systems of ladderane lipids and their mechanisms have attracted much attention from organic and theoretical chemists, biochemists, and structural biologists. The gene clusters of type II fatty acid biosynthesis (FASII) of a model species of anammox bacteria (*Kuenenia stuttgartiensis*) contain several genes encoding enzymes that are not typically involved in FASII, such as *S*-adenosyl-l-methionine (SAM)-dependent methyltransferases (SAM-MTs), radical SAM enzymes, and enzymes similar to phytoene desaturase [14]. Comparative analyses of metagenomic data of *K. stuttgartiensis* and other data suggested that the biosynthetic intermediates required to produce the ladder-like moiety were connected to acyl carrier protein (ACP) via the phosphopantetheine group and the SAM-dependent and other enzymes encoded in anammox FASII gene clusters process the intermediates to produce ladderane lipids, and such process have been observed for canonical FASII and type I and II polyketide synthase (PKS) systems, where the biosynthetic intermediates connected to ACP through the phosphopantetheine group are elongated and modified by several enzymes or enzyme modules [14]. The pure cultivation of anammox bacteria has not yet been achieved, and the growth rate of these bacteria is notably low; thus, identifying the responsible genes by conventional genetic analyses of ladderane biosynthesis systems, such as gene knock-out experiments, is nearly impossible. To elucidate the ladderane lipid biosynthetic pathway, codon-optimized candidate ladderane biosynthesis genes were investigated by heterologous expression in *Escherichia coli* [15]. However, the production of ladderane lipids or its biosynthetic intermediates in this system was not successful. To the best of our knowledge, no direct experimental evidence on the biosynthesis mechanism of ladderane lipids has been reported.

Two SAM-MTs (AmxMT1, 2) are found in many species and genera of anammox bacteria. AmxMT1 is categorized as a class I SAM-MT, which has a Rossmann fold and is the largest family of SAM-MTs, and the enzyme is unusual because it is phylogenetically distant from homologous SAM-MTs in this family [14]. In this study, we focused on AmxMT1 from the anammox bacteria species *Brocadia fulgida,* which contains a relatively larger amount of ladderane lipids than other species of anammox bacteria [16], and determined the crystal structure at 1.69-Å resolution. To explore whether AmxMT1 is involved in the biosynthesis of unusual lipids in anammox bacteria, we performed a pull-down assay to examine the interaction between AmxMP1 and ACP from anammox bacteria (AmxACP) and constructed AmxMT1/AmxACP complex models.

## 2. Results and Discussion

### 2.1. Overall Structure of Amx MT1 and Cosubstrate Binding Mode

**Figure 2:**
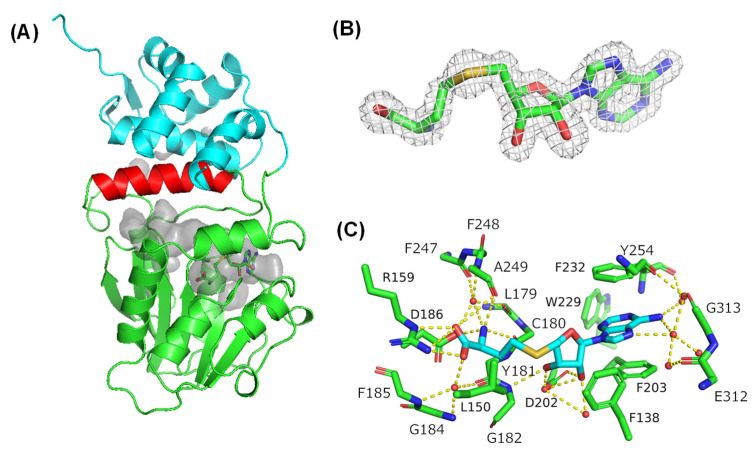
Crystal structure of SAM-dependent methyltransferases from the anammox bacteria (AmxMT1) *Brocadia fulgida* along with *S*-adenosyl-l-homocysteine (SAH). (**A**) Overall structure of AmxMT1. α-domain at the N-terminus, αβα sandwich core domain, and inserted α9 are shown in cyan, green, and red, respectively. SAH is shown as a stick model. A substrate binding and active site pocket is shown in gray as a surface model. (**B**) *F*_o_ − *F*_c_ Polder omit electron density map for SAH contoured at 3.0 σ. (**C**) Close-up view revealing the SAH binding mode. The main chain amino acid residues and their numbers are underlined.

We determined the crystal structure of AmxMT1 by single-wavelength anomalous dispersion (SAD) phasing at 1.60-Å resolution (Table S1), and one molecule was found in the asymmetric unit. AmxMT1 has an NAD(P)-binding Rossmann fold similar to the αβα sandwich core domain, which consists of seven β-strands and five α-helices and is commonly found in class I SAM-MTs (Figure 2A) [17,18]. An α-helix (α9) was inserted between β-strand 7 and α-helix 10 of the core domain. The core domain and α9 are covered by an additional α-domain at the N-terminus, and *S*-adenosyl-l-homocysteine (SAH) is located in the pocket between the core domain and inserted helix, although we added SAM to the enzyme for crystallization (Figure 2A,B). The ribosyl moiety of the bound SAH forms hydrogen bonds with D202, a water molecule, and the main-chain NH of G182. The terminal carboxyl group of SAH forms a salt bridge with R159 and a hydrogen-bond network that includes a water molecule and several main-chain atoms (carbonyl groups of Y181 and A249; and NH of G184 and F185). Additionally, the terminal amino group of SAH forms a hydrogen-bounding network that includes a water molecule, the D186 sidechain, and several main chain atoms (carbonyl groups of F247 and A249; NH of F248). The D186 side chain and mainchain carbonyl groups of C180 and Y181 are hydrogen-bonded to the amino group of SAH. The adenine ring is surrounded by aromatic side chains F138, F203, F232, Y254, and W229, and its edge is exposed to the molecular surface. Although the SAM-binding residues are very weakly conserved in class I SAM-MTs, salt-bridge and hydrogen-bonding interactions between the co-substrate and AmxMT1 are similar to those found in other class I SAM-MTs [17].

### 2.2. Comparison with Other SAM-MTs

**Figure 3:**
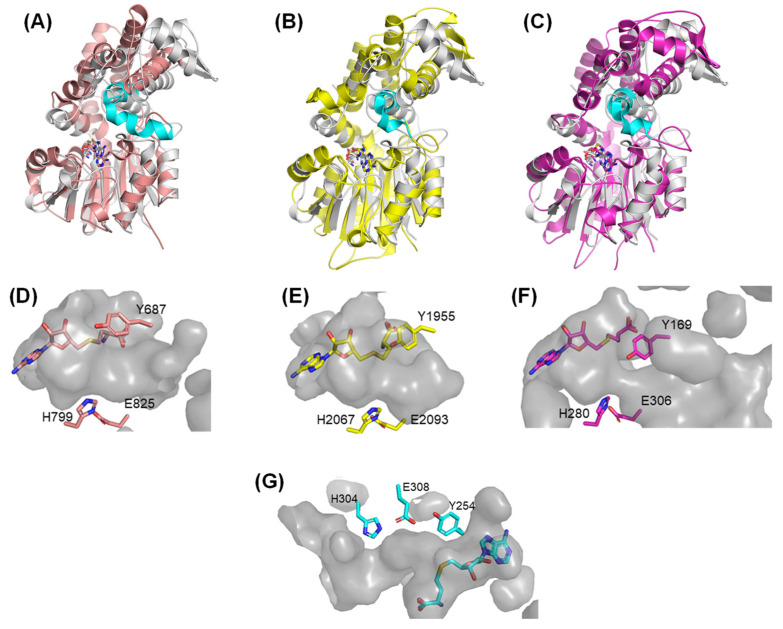
Structural comparison of SAM-dependent methyltransferases from anammox bacteria (AmxMT1) with C-methyltransferase (MT) domains. Overlaid structure of AmxMT1 (gray) with PsoF C-MT (pink, **A**), citrinin synthase CMeT (yellow, **B**), and CurJ C-MT (magenta, **C**) domains. The α-helices inserted to the core domains of PsoF C-MT, citrinin synthase CMeT, and CurJ C-MT are shown in cyan. A catalytic dyad and Tyr residue that facilitates methyl transfer reaction in the substrate binding and active site of (**D**) PsoF C-MT, (**E**) citrinin synthase CMeT, (**F**) CurJ C-MT, and (**G**) AmxMT1 are shown as stick models.

A similar overall structure, the typical class I MT core structure with the inserted α-helix and an additional N-terminal domain, was also found for the *C*-methyltransferase domain of PsoF (PsoF *C*-MT; RMSD 2.68 Å) (Figure 3A), which is involved in pseurotin A biosynthesis by a polyketide synthase and non-ribosomal peptide synthetase hybrid enzyme, PsoA [19]. Citrinin synthase CMeT and CurJ *C*-MT, both of which are MT domains of PKS, also adopt similar overall structures (Figure 3B,C; RMSD CuJ *C*-MT: 3.56 Å and citrinin synthase CMeT: 3.80 Å) [20,21]. These *C*-MT domains have a long pocket and SAM between the core domain and the inserted α-helix, suggesting that a long β-ketoacyl substrate is connected to the carrier proteins (thiolation domain of PsoA; ACP domains of CurJ and citrinin synthase) and binds to the pocket, and the methyl group of SAM is transferred to the substrate, which is assisted by the His-Glu catalytic dyad and Tyr side chain located at the surface of the substrate binding pocket (Figure 3D–F). In the crystal structure of AmxMT1, a similar long pocket and SAH were found between the core domain and inserted helix α9. A His-Glu dyad (H304-E308) and Y254 were also found on the surface of the pocket (Figure 3G) [20]. The position of SAH in AmxMT1 is similar to that of SAM or SAH found in many other class I SAM-MTs, including PsoF *C*-MT, CurJ *C*-MT, and citrinin synthase CMeT. The presence of the long pocket, His-Glu dyad, and Y254 in AmxMT1, as observed for other PKS *C*-MTs, suggests that a linear β-ketoacyl substrate binds to this pocket.

### 2.3. ACP/C-MT and AmxACP/AmxMT1 Complex Models

**Figure 4:**
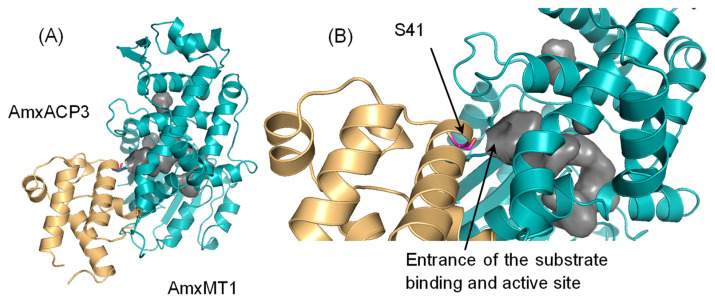
AmxACP3-AmxMT1 complex model (**A**) AmxACP3-AmxMT1 from Brocadia fulgida binary complex model predicted by AlphaFold. AmxACP3 and AmxMT1 are shown in light orange and teal, respectively. Ser41 of AmxACP3, to which the substrate would be connected, is shown as a magenta stick model. A substrate binding pocket of AmxMT1 is shown in gray as a surface model. (**B**) Close-up view of the AmxACP3 and AmxMT1 interface region in the model complex.

The substrates for CurJ *C*-MT and citrinin synthase CMeT are anchored to ACP; thus, these enzymes form a relatively stable complex with ACP, as observed for ACP complexed with a β-ketoacyl-ACP synthase in the FASII system [22,23]. The recently developed AlphaFold is a novel machine-learning-based program that can predict protein structures from amino acid sequences [24]. AlphaFold can even predict near-native protein–protein complex structures from sequences in many cases, although the program fails to predict antibody-antigen complex structures [25]. Indeed, the *E. coli* ACP/β-ketoacyl-ACP synthase and anammox (*B. fulgida*) ACP/β-ketoacyl-ACP synthase (encoded by *BROFUL01968* and *01969* genes, respectively) complex structures predicted by AlphaFold are very similar to the *E. coli* ACP/β-ketoacyl-ACP synthase complex crystal structure (RMSD 0.379 (*E. coli* to *E. coli*) and 0.875 (*B. fulgida* to *E. coli*) Å; Figure S1), suggesting that AlphaFold can reliably predict ACP and its partner enzyme complex structure. Next, we constructed the CurJ *C*-MT/ACP and citrinin synthase CMeT/ACP complex models (Figure S2). Although the crystal structures of these complexes have not yet been determined, the methyltransferase moiety structures of the complex model are very similar to the crystal structures. Furthermore, the quaternary structures of these complexes are consistent with the plausible binding mode, in which their substrate connected to ACP binds to the substrate binding pocket in the methyltransferases. In the model complex structures, Ser side chains of ACP (Ser38 of ACP for citrinin synthase CMeT; Ser17 of ACP for CurJ *C*-MeT), where the substrates are loaded through the phosphopantetheine group [22,26], were located close to the entrance to the substrate binding and active site pocket of the *C*-MTs, implying that the model complex structures are reasonable and potentially reliable. Finally, we chose AmxACP3 (encoded by *BROFUL02173* gene) from three AmxACPs and constructed a model of AmxACP3/AmxMT1 from the anammox bacteria *B. fulgida* (Figure 4) based on the following reasons: first, among the three AmxACPs found in anammox FASII gene clusters, AmxACP3 is the least homologous to canonical ACPs in the bacterial FASII system and is expected to be involved in the biosynthesis of unusual fatty acids; second, crystallographic analysis and molecular dynamics simulation of AmxACP3 revealed unique structural features, including the functionally important helix (α3) adopting a rare six-residue-long flexible 3_10_-helix conformation and a conserved two consecutive Phe motif that would shield the thioester linkage between the protein’s prosthetic group (phosphopantetheine) and its acyl cargo [27]. The structures of AmxACP3 and AmxMT1 in the predicted complex model were very similar to their respective X-ray crystal structures. In the complex model, Ser41 of AmxACP3, which would anchor a substrate through the phosphopantetheine group, is located close to the entrance of the plausible substrate binding pocket and active site of AmxMT1 (Figure 4B), as previously observed for the CurJ *C*-MT/ACP and citrinin synthase CMeT/ACP complex models. Similar complex structures were also predicted for AmxACP3/AmxMT1 from the anammox bacteria *K. stuttgartiensis* and *Jettenia caeni* (Figure S3). Therefore, the quaternary structure of the complex model is consistent with the hypothesis that the substrate connected to AmxACP3 binds to the pocket of AmxMT1.

### 2.4. AmxMT1 Pull-Down Assay

**Figure 5:**
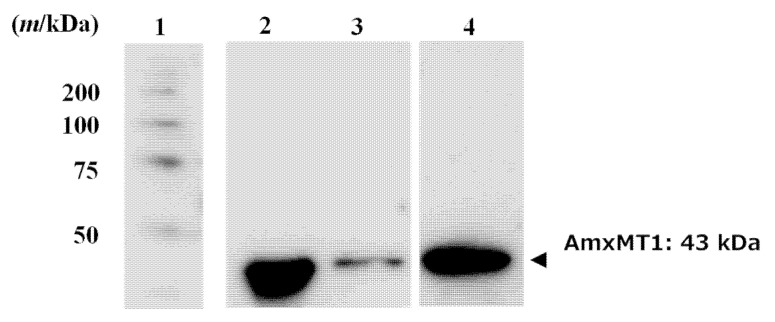
AmxACP3 pull-down assay to show the interaction between AmxACP3 and AmxMT1. AmxMT1 was detected by Western blotting with anti-His tag mouse IgG and HRP-linked anti-mouse goat IgG. Lane 1: Protein molecular weight marker. Lane 2: Supernatant of cell-free extract of *E. coli* that produced AmxMT1. Lane 3: Eluate from AmxACP3 free-Sepharose^TM^ resin. Lane 4: Eluate from AmxACP3-immobilized Sepharose^TM^ resin in a pull-down assay.

To confirm whether AmxACP3 interacted with AmxMT1, we prepared AmxACP3 immobilized resin and performed a pull-down assay. ACP synthase (ACPS) binds to ACPs in the FASII system to produce holo-ACPs [22,28]. The AmxACP3-immobilized resin indeed bound AmxACP synthase (AmxACPS; encoded by *BROFUL00292* gene) from *B. fulgida*, indicating that the immobilized AmxACP3 could bind their partner enzymes (Figure S4). The pull-down assay was then performed on the lysate supernatant of *E. coli* expressing AmxMT1 fused with the His-tag using AmxACP3-immobilized resin. Western blotting following the pull-down assay using anti-His-tag antibodies revealed that the amount of AmxMT1 bound to the immobilized AmxACP3 is remarkably larger than that non-specifically bound to the resin (Figure 5), indicating that AmxMT1 interacts specifically with AmxACP3. This observation is consistent with the AmxACP/AmxMT1 complex structure model and the notion that the β-ketoacyl group loaded onto AmxACP3 is a potential substrate of AmxMT1.

### 2.5. Potential Substrate Binding Mode and Reaction Mechanisms of AmxMT1

**Figure 6:**
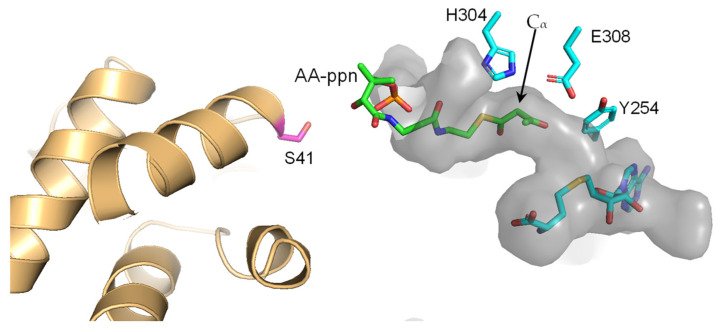
Potential binding orientation of acetoacetyl phosphopantetheine (AA-ppn) in AmxMT1. A substrate binding pocket of AmxMT1 is shown in gray as surface model. Putative catalytic key residues, SAH, and AA-ppn binding model obtained by docking simulation are shown as stick models. AmxACP3 in the predicted model of Brocadia fulgida AmxACP3-AmxMT1 is shown in light orange. Ser41 of AmxACP is shown as a magenta stick model. The C_α_ atom of AA-ppn is indicated by an arrow.

Citrinin synthase CMeT has a relatively wide substrate binding pocket, and the enzyme can accommodate a long substrate, tetraketide loaded onto ACP, likely with a bent conformation [21]. Computational docking of an analog of a plausible natural substrate of PsoF *C*-MT indicated that the substrate binding pocket of this enzyme is deep enough to accommodate the substrate. Furthermore, CurJ *C*-MT has a long substrate binding tunnel that is parallel to the SAM, and substrate modelling suggests that the natural long substrate can bind to the tunnel [20]. Therefore, the shape and volume of the substrate binding pocket in the crystal structure of AmxMT1 allow us to speculate on a potential substrate. The substrate binding pocket of AmxMT1 is shorter than that of CurJ *C*-MT and narrower than that of citrinin synthase CMeT, implying that the substrate of AmxMT1 has a short chain connected to AmxACP3 through the phosphopantetheine group. Therefore, we performed a docking simulation of acetoacetyl phosphopantetheine (AA-ppn), which is the shortest β-ketoacyl phosphopantetheine (Figure 6). Docking simulation results indicated that the substrate binding pocket of AmxMT1 could accommodate AA-ppn. A plausible catalytic dyad of AmxMT1, H304-E308, is located close to the C_α_ atom of the bound AA-ppn. Thus, a small linear substrate like AA-ppn or others connected to AmxACP3 would be methylated at C_α_ as seen for the reactions by other PKS *C*-MTs.

A linear molecule with many cyclopropane rings (oligo-cyclopropane) is a potential precursor for constructing the ladder moiety of ladderane lipids based on quantum chemical calculations (Figure S5) [29]. Enzymes such as cyclopropane fatty acid (CFA) synthases and cyclopropane mycolic acid (CMA) synthases can produce cyclopropane rings from unsaturated lipids, and these enzymes are homologous to SAM MT family members [30,31]. Several X-ray crystal structures of CFA synthases from *E. coli* [32], *Aquifex aeolicous* (PDBID: 7QOS), *Lactobacillus acidophilus* [33] and CMA synthases (CmaA1, Cma2, and PcaA) [34] have been reported. In these crystal structures, bicarbonate was found at the active site, and the amino acid residues consisting of bicarbonate binding sites (e.g., H167, E140, and Y232 in CmaA1) were strictly conserved [35]. In the reactions catalyzed by these enzymes, the methyl group derived from SAM is electrophilically transferred to the substrate, unsaturated lipid, to form a methylated intermediate cation, and then the bound bicarbonate abstracts a proton from the cationic intermediate with assistance from a Glu residue (E140 in CmaA1) to produce a cyclopropane ring [35]. As shown by biochemical experiments, the bound bicarbonate and its binding amino acid residues are essential for enzymatic cyclopropane ring formation [35]. CFA synthases and CMA synthases also adopt an overall structure with two domains, as seen for AmxMT1; however, bicarbonate and the bicarbonate binding site were not found in the crystal structure of AmxMT1, suggesting that AmxMT1 cannot produce a cyclopropane ring in the same manner as CFA synthases and CMA synthases.

Although the crystal structure of AmxMT1 cannot explicitly predict its natural substrate, the substrate binding and active site pocket similarity between AmxMT1 and PKS *C*-MTs and plausible complexation between AmxMT1 and AmxACP3 suggest that a small linear β-ketoacyl group connected to AmxACP3 through phosphopantetheine would be a substrate for AmxMT1 and the enzyme methylates the C_α_ atom of the β-ketoacyl group, as seen for PKS *C*-MTs.

### 2.6. Conclusions

Ladderane lipids from anammox bacteria have an unprecedented ladder-like structure that consists of up to five linearly concatenated four-membered rings. The mechanisms underlying the construction of such highly strained carbon skeleton in anammox bacteria remain to be fully established. Although no direct experimental data have been reported showing the biosynthetic construction mechanism of the ladder-like skeleton, Rattray et al. proposed that biosynthetic intermediates used in the production of a ladder-like structure would be connected to AmxACP and atypical FASII enzymes, such as SAM MTs, radical SAM enzymes, and others encoded by FASII gene clusters from anammox bacteria, process the intermediate to produce the ladder-like structure. For the first time, we determined the crystal structure of AmxMT1 from an anammox bacteria species, *B. fulgida*. As expected from an AmxMT1/AmxACP3 complex model predicted by AlphaFold, AmxMT1 exhibited a specific interaction with AmxAPC3, which was substantiated by an AmxACP pull-down assay of AxmMT1. A comparison of the crystal structure of AmxMT1 with those of PKS *C*-MTs and docking simulations suggested that a linear small β-ketoacyl group connected to AmxACP3 would be a substrate of this enzyme and its C_α_ atom is likely methylated to produce a branched lipid. Given the limited size of the substrate binding pocket of AmxMT1, the enzyme would not be able to construct the ladder skeleton of the ladderane lipids in this pocket. Moreover, the enzymes responsible for constructing the ladder structure of ladderane lipids have not been identified. However, the specific interaction between AmxACP3 and AmxMT1, which is not typically involved in FASII, is consistent with the hypothesis that a precursor connected to ACP would be processed by enzymes encoded in anammox FASII gene clusters, such as radical SAM enzymes. To reveal the biosynthetic pathway of ladderane lipids, the FASII gene clusters of anammox bacteria would have to be reconstituted in other microorganisms. Finally, this study encourages further research on the biosynthesis of the highly strained carbon skeleton in ladderane lipids, and we hope that the mechanism underlying the construction of the ladder structure in anammox bacteria will be clarified in the near future.

## 3. Materials and Methods

### 3.1. Protein Production and Purification

To enhance protein production in *E. coli*, the complete coding sequences of the proteins used in this study were codon optimized. The TEV protease recognition site and a 6 × His tag were added to the C-terminus of AmxMT1. The TEV protease recognition site and 8 × His-tag were added to the C-terminus of AmxACP3. The N-terminus of AmxMT1 was predicted to be disordered; thus, five residues at the N-terminus were removed. The genes encoding AmxACP3, AmxMT1, and AmxACPS were inserted into pET28b by seamless cloning to create pAmxACP3, pAmxMT1, and pAmxACPS, respectively. *E. coli* BL21(DE3) was transformed with pACP3 and pACPS, respectively, and grown in LB medium with 25 μg/mL kanamycin, 0.025 % glucose, and 500 μM IPTG at 30 °C. After 16–24 h shaking, cells were harvested and stored at −80 °C until further use. *E. coli* C43(DE3) was transformed with pAmxMT1 and pGro7 chaperon co-expressing plasmid and grown in LB medium with 25 μg/mL kanamycin, 30 μg/mL chloramphenicol, 0.026 % arabinose, 0.035 % glucose, and 500 μM IPTG at 25 °C. After 16 h shaking, cells were harvested and stored at −80 °C until further use.

Cells that produced AmxACP3 were resuspended in ACPA buffer (50 mM potassium phosphate pH 8.0, 200 mM NaCl, 500 μM DTT, and 20 mM imidazole). Cell suspensions were treated with 2 mM PMSF, 0.5 mg/mL lysozyme, 6 μg/mL Sm2 nuclease, and 0.5% Triton X-100 using a rotator for 30 min at 4 °C, and clarified by centrifugation at 10,000× *g* for 10 min at 4 °C. An equal volume of 2-propanol was added to the supernatant, which was then cleared by centrifugation at 10,000× *g* for 10 min at 4 °C. The supernatant was diluted with 2-fold ACPA buffer and centrifuged at 10,000× *g* for 10 min at 4 °C. The supernatant was applied to an immobilized-Ni-NTA agarose column (QIAGEN, Hilden, Germany) pre-equilibrated with ACPA buffer, and ACPB buffer (50 mM potassium phosphate pH 8.0, 200 mM NaCl, 500 μM DTT, and 250 mM imidazole) was used to elute AmxACP3. The fractions containing AmxACP3 were collected and concentrated to a volume of 10 mL or less using an Amicon^®^ Ultra 3 K (Merck Millipore, Darmstadt, Germany). The concentration of AmxACP3 was estimated using the absorbance and theoretical extinction coefficient at 280 nm. TEV protease (10 % weight of AmxACP3) was added and dialyzed against 1 L of ACPC buffer (50 mM potassium phosphate pH 8.0, 200 mM NaCl, 500 μM DTT). The cleaved 8 × His tag and protease were removed using immobilized Ni affinity chromatography.

Cells that produced AmxMT1 were resuspended in AmxMT1A buffer (50 mM Tris-HCl pH 8.0, 100 mM NaCl, 10 % glycerol, and 20 mM imidazole). PMSF (final concentration 2 mM), small amounts of DNase I, and lysozyme were added to the cell suspension. The cell suspensions were sonicated and cleared by centrifugation at 10,000× *g* for 10 min at 4 °C. The supernatant was subjected to immobilized Ni affinity chromatography using AmxMT1A buffer and AmxMT1 was eluted with AmxMT1B buffer (50 mM Tris-HCl pH 8.0, 100 mM NaCl, 10 % glycerol, and 250 mM imidazole). The eluates containing AmxMT1 were concentrated to a volume of 10 mL or less using an Amicon^®^ Ultra 30 K. To cleave 6 × His tag, an equal amount of TEV protease was added and dialyzed against AmxMT1C buffer (50 mM Tris-HCl pH 8.0, 100 mM NaCl, 10 % Glycerol). The cleaved 6 × His tag and protease were removed by immobilized Ni-affinity chromatography. AmxMT1 was further purified by a Superdex 75 size-exclusion column (Cytiva, Tokyo, Japan) using AmxMT1C buffer.

### 3.2. Crystallization, Data Collection, and Structure Determination of AmxMT1

AmxMT1 solution was concentrated to 10 mg/mL using Amicon^®^ Ultra 30 K in AmxMT1C buffer containing 5 mM DTT and 2.25 mM SAM. Native crystals of AmxMT1 were grown by mixing 1 μL of AmxMT1 buffer with an equal volume of mother liquor (0.1 M HEPES pH 7.0, 15% polyethylene glycol 2000) by the sitting drop vapor diffusion method at 20 °C. Native crystals were soaked in the solution containing 250 mM sodium acetate, 25% polyethylene glycol 3350, 10% glycerol, 10 mM dithionite, and a saturated concentration of selenourea to prepare selenourea-bound AmxMT1 crystals [36]. The native and selenourea-bound AmxMT1 crystals were flash-frozen using a cryo-stream. The SAD data of selenourea-bound AmxMT1 crystals were collected at 0.9794 Å using beamline BL32XU at SPring-8. To solve the structure of AmxMT1 from the SAD data set using Se anomalous scattering, the program Phenix was used for heavy-atom search, phase calculation, density modification, and initial model building. The high-resolution structure was determined by a molecular replacement method using the model obtained by SAD phasing as a search model. Model building and refinement were performed using Coot and Phenix refine, respectively [37,38]. The data collection and refinement statistics are shown in Table S1.

### 3.3. Preparation of ACP-Immobilized Sepharose and Pull-Down Assay

AmxACP3-immobilized Sepharose^TM^ resin was prepared by adding 140 μg AmxACP3 in coupling buffer (0.2M NaHCO_3_ and 500 mM NaCl) to 100 μL of NHS-activated Sepharose^TM^ 4 fast flow (GE Healthcare Life Sciences, Tokyo, Japan). After the coupling reaction, the uncoupled NHS group was blocked using a blocking buffer (500 μM ethanolamine pH 8.3 and 500 mM NaCl). The ACP-immobilized Sepharose^TM^ resin was washed three times with five column volumes of PBST buffer (10 mM sodium phosphate, pH 7.5, 0.15 M NaCl, and 0.05% tween-20) and stored in PBS buffer (10 mM sodium phosphate, pH 7.5, and 0.15 M NaCl) with 0.05 % sodium azide. Protein concentration was estimated using a BCA assay.

*E. coli* cells that produced ACPS or AmxMT1 were resuspended in a pull-down buffer (20 mM potassium phosphate, pH 8.0, 200 mM NaCl, 500 μM DTT, and 10 % glycerol). The cell suspensions were treated with 2 mM PMSF, 0.5 mg/mL lysozyme, 6 μg/mL Sm2 nuclease, and 0.5 % Triton X-100 at 4 °C with rotating for 1 h and cleared by centrifugation. The supernatant containing AmxMT1 or AmxACPS (500 µL) was added to 25 μL of AmxACP3-immobilized Sepharose^TM^ and incubated at 4 °C overnight using a rotator. The Sepharose^TM^ resin was collected by mild centrifugation and washed twice with 500 μL pull-down buffer. The proteins bound to the resin were released by boiling at 95 °C in 50 μL of 2×SDS sample buffer, analyzed by SDS-PAGE, and subjected to CBB staining or Western blotting. To detect 6×His tagged AmxMT1, Western blotting was performed using anti-His mouse IgG and anti-IgG (H + L chain) (mouse) pAb-HRP (MBL Life Science, Tokyo, Japan).

### 3.4. Potential Substrate Docking Simulation of AmxMT1

The model structure of *S*-acetoacethyl-4′-phosphopantetheine was refined by MM2 processing in Chem3D 16.0 (Perkin Elmer) and partially restrained by AutoDockTools-1.5.6. Hydrogen atoms were added and SAH was removed from the crystal structure of AmxMT1 to prepare the receptor model for the docking simulations of *S*-acetoacethyl-4′-phosphopantetheine, which was performed using Webina 1.0.3. The search box (15 × 8 × 15 Å^3^) was set to prevent ligands from clashing with SAH and exhaustiveness was set to 128.

## Data Availability

The atomic coordinates and the structure factors for AmxMT1 (PDB ID 8HD2) have been deposited in the Protein Data Bank (www.rcsb.org, accessed on 14 November 2022).

## Supplementary Materials

The following supporting information can be downloaded at https://www.mdpi.com/article/10.3390/ijms24010744/s1.
