# Supplementary material for: Anammox Bacterial S-Adenosyl-l-Methionine Dependent Methyltransferase Crystal Structure and Its Interaction with Acyl Carrier Proteins
Source: Int J Mol Sci. 2023 Jan 1;24(1):744. doi: 10.3390/ijms24010744 (PMC9821293; doi:10.3390/ijms24010744)
Supplement: Supplementary file 1 [file ijms-24-00744-s001.zip › ijms-2094413-supplementary.pdf]

Table S1. Data collection and refinement statistics.

	Se-SAD data	High resolution data
**Data collection**		
Beam source	BL32XU (SPring8)	BL32XU (SPring-8)
Wavelength (Å)	0.9794	1.0000
Resolution range	49.20−1.98 (2.05−1.98)	45.12−1.60 (1.66−1.60)
Space group	*P*1	*P*1
Cell dimensions		
a, b, c (Å)	35.09 47.53 49.73	35.02 48.06 49.83
α, β, γ (°)	82.86 84.24 70.12	82.59 84.12 70.67
Total reflections	1616644	131492
Unique reflections	40436 (6484)[a]	38897 (1761)[a]
Multiplicity	40.0 (40.6)[a]	3.4 (3.5)[a]
Completeness (%)	100 (100)[a]	97.13 (95.12)[a]
Mean I/sigma(I)	13.05 (4.05)[a]	7.0 (1.5)[a]
R_{meas} (%)[b]	23.4 (82.2)[a]	10.3 (62.0)[a]
$CC_{1/2}$[c]	0.997 (0.948)[a]	0.994 (0.761)[a]
**Refinement**		
Reflections used in refinement		38856 (3800)[a]
Reflections used for R_{free}		1881 (185)[a]
R_{work}		0.1802 (0.2128)[a]
R_{free}		0.2080 (0.2337)[a]
Number of non-hydrogen atoms		2870
Macromolecules		2597
Ligands		0
Solvent		273
Protein residues		315
RMS(bonds)		0.006
RMS(angles)		0.80
Ramachandran favoured (%)		99.35
Ramachandran allowed (%)		0.65
Ramachandran outliers (%)		0.00
Rotamer outliers (%)		0.37
Clash score		2.36
Average *B*-factor		23.64
Macromolecules		22.49
Solvent		34.59

[a] Numbers in parentheses were calculated from data for the highest resolution shell.

[b] $R_{meas} = \Sigma_{hkl} \{N(hkl)/[N(hkl)-1]\}^{1/2} \Sigma_i |I_i(hkl) - \langle I(hkl)\rangle| / \Sigma_{hkl}\Sigma_i I_i(hkl)$.

[c] Correlation coefficient between intensities from random half-data sets.

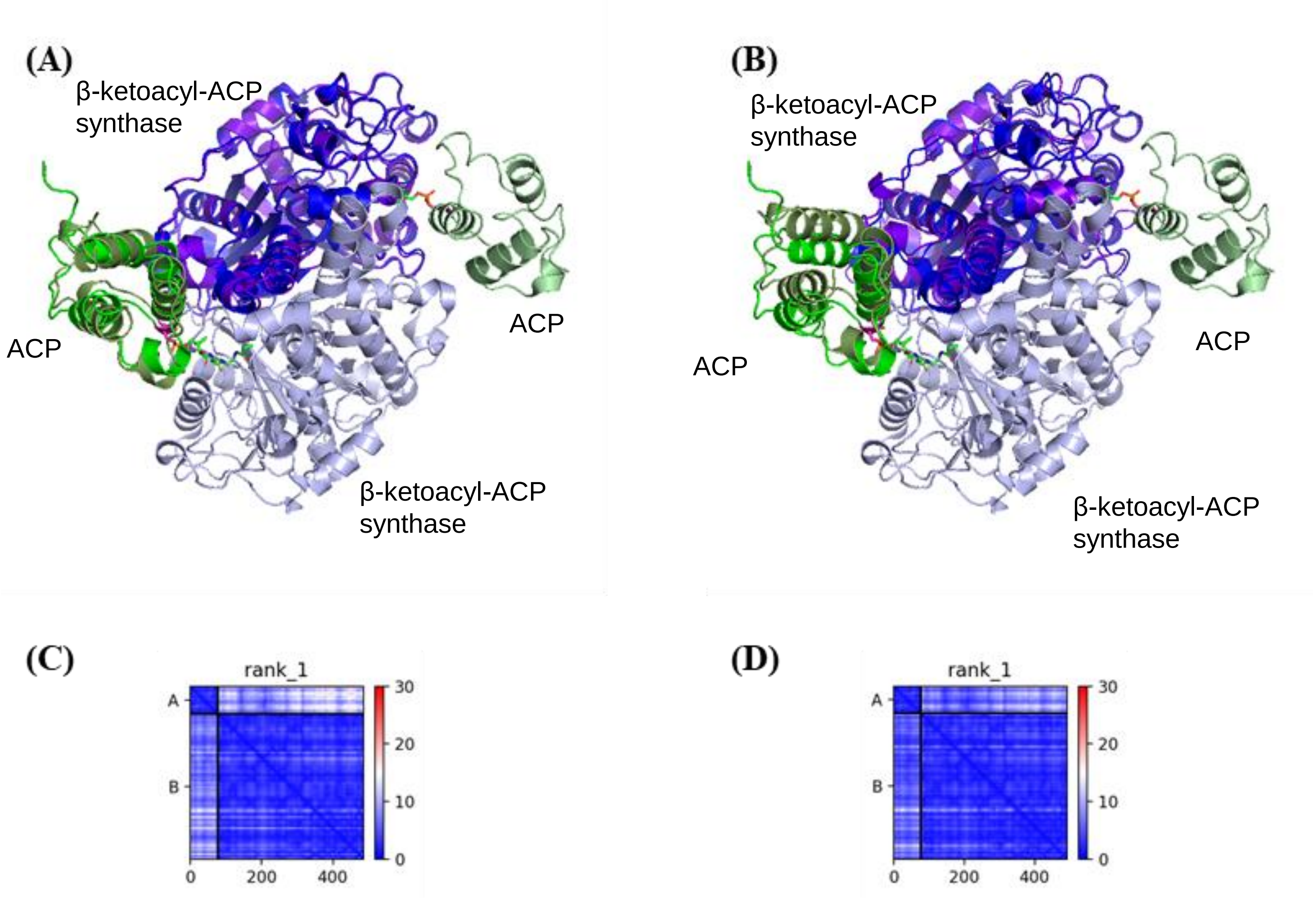

Figure S1. β-ketoacyl-ACP synthase-ACP complex model predicted by AlphaFold and crystal structure of β-ketoacyl-ACP synthase-ACP complex (A) Crystal structure of the *Escherichia coli* (β-ketoacyl-ACP synthase)$_2$-ACP$_2$ (dimer of heterodimer) complex is overlaid with the *E. coli* β-ketoacyl-ACP synthase-ACP complex model (monomer of heterodimer) predicted by AlphaFold. ACP chains A and B and β-ketoacyl-ACP synthase chains A and B of the crystal structure are shown in pale green, green, light blue, and blue, respectively. ACP and β-ketoacyl-ACP synthase of the complex model are shown in smudge and purple-blue, respectively. (B) Crystal structure of *E. coli* (β-ketoacyl-ACP synthase)$_2$-ACP$_2$ (dimer of heterodimer) complex is overlaid with the *Brocadia fulgida* β-ketoacyl-ACP synthase-ACP complex model (monomer of heterodimer) predicted by AlphaFold. *B. fulgida* ACP and β-ketoacyl-ACP synthase of the complex model are shown in smudge and purple-blue, respectively. (C) Predicted alignment errors of the *E. coli* β-ketoacyl-ACP synthase-ACP complex model. (D) Predicted alignment errors of the *B. fulgida* β-ketoacyl-ACP synthase-ACP complex model.

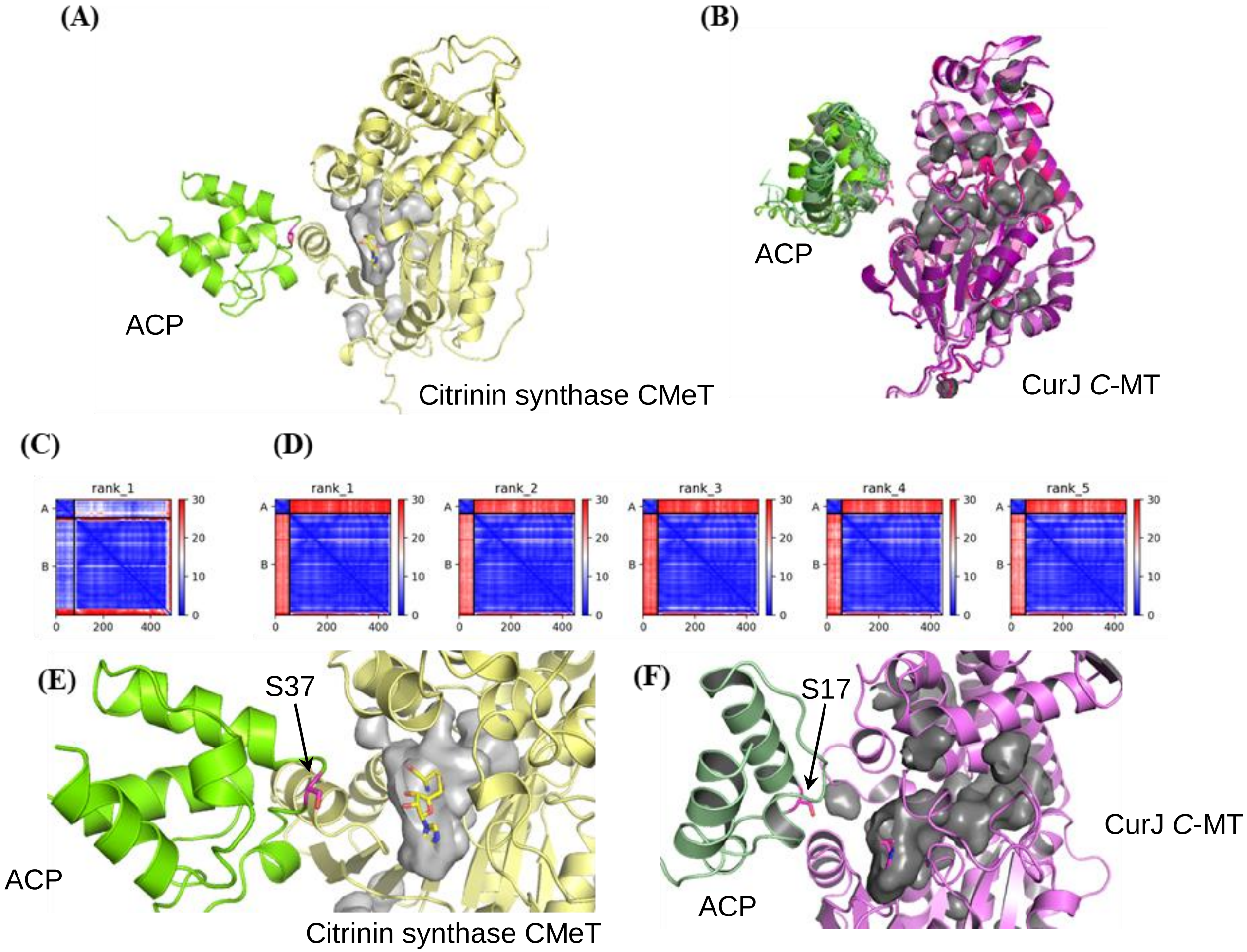

Figure S2. ACP-PKS *C*-MT complex models predicted by AlphaFold A) Citrinin synthase ACP-CMeT complex model predicted by AlphaFold. ACP and citrinin synthase CMeT in a predicted complex model are shown in chartreuse and pale yellow, respectively. A substrate binding pocket of the crystal structure of citrinin synthase CMeT is shown as surface model. (B) CurJ *C*-MT-ACP complex model predicted by AlphaFold. Five predicted models of ACP-CurJ *C*-MT complex are overlaid. A substrate binding pocket of the crystal structure of CurJ *C*-MT is shown as the surface model. (C) Predicted alignment errors of the citrinin synthase ACP-CMeT complex model. (D) Predicted alignment errors of five CurJ *C*-MT-ACP complex models. (E) Close-up view of the citrinin synthase ACP-CMeT complex model. ACP and citrinin synthase CMeT are shown in chartreuse and pale yellow, respectively. A substrate binding pocket of the crystal structure of citrinin synthase CMeT is shown as the surface model. Ser37 of ACP is shown as a magenta stick model. (F) Close-up view of the ACP-CurJ *C*-MT complex model of rank 3. ACP and CurJ *C*-MT are shown in pale green and violet, respectively. A substrate binding pocket of the crystal structure of citrinin synthase CMeT is shown as the surface model. Ser17 of ACP is shown as a magenta stick model.

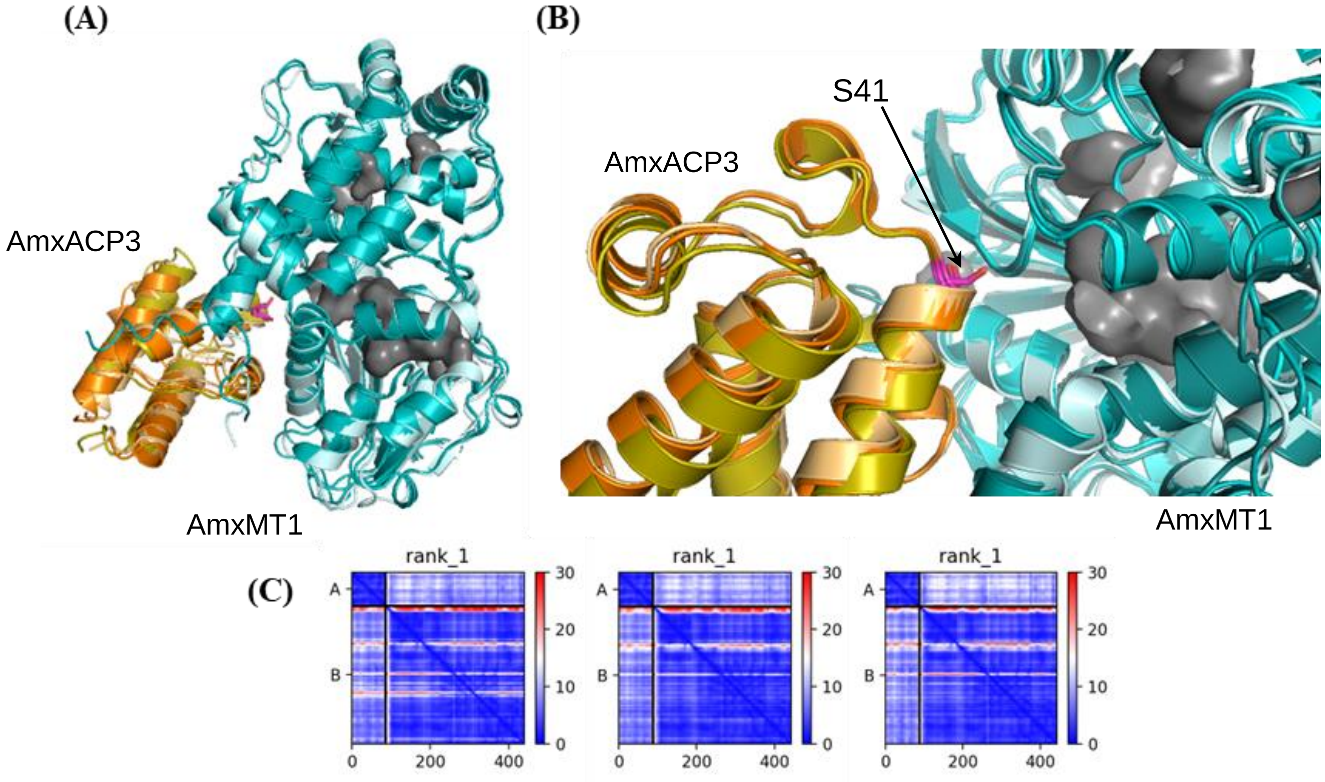

Figure S3. AmxACP3-AmxMT1 complex models predicted by AlphaFold (A) Overall structure of the AmxACP3-AmxMT1 complex models from *Kuenenia stuttfartiensis* (orange and pale cyan), *Brocadia fulgida* (light orange and teal), and *Jettenia caeni* (olive and deep teal). (B) Close-up view of the AmxACP3 and AmxMT1 interface region in the model complex. (C) Predicted alignment errors of the AmxACP3-AmxMT1 complex models from *K. stuttgartiensis* (left), *B. fulgida* (middle), and *J. caeni* (right).

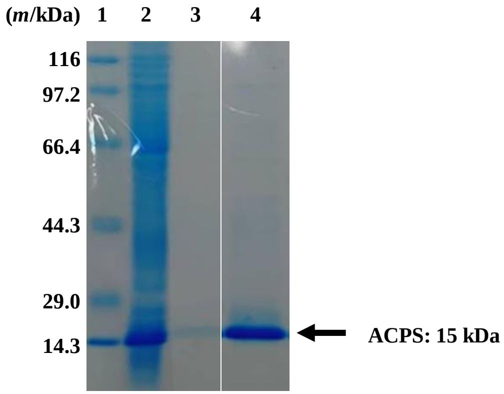

Figure S4. AmxACP3 Pull-down assay to show the interaction between AmxACP3 and ACP synthase from anammox bacteria (AmxACPS). Lane 1: Protein molecular weight markers. Lane 2: Supernatant of cell free extract of *Escherichia coli* that produced AmxACPS. Lane 3: Eluate from AmxACP3-free Sepharose resin. Lane 4: Elute from AmxACP3-immobilized Sepharose resin. Proteins are stained by Coomassie brilliant blue.

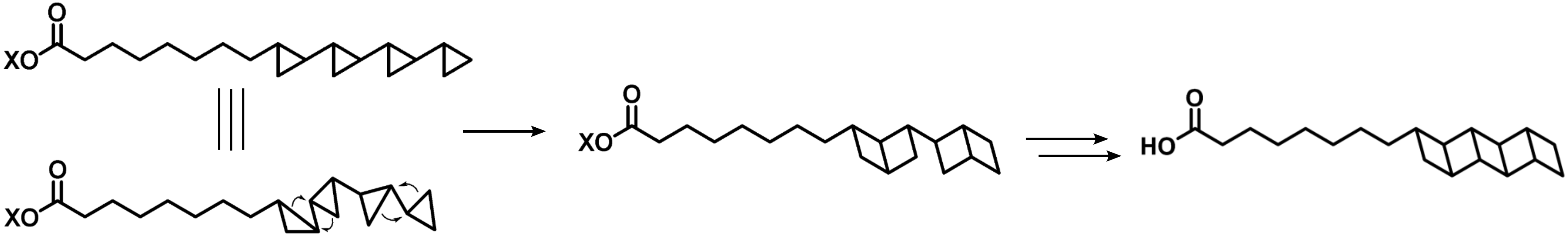

Figure S5. Potential biosynthetic pathway to produce ladderane fatty acid from oligo-cyclopropane precursors
